# EEG functional connectivity is sensitive for nitrogen narcosis at 608 kPa

**DOI:** 10.1038/s41598-022-08869-8

**Published:** 2022-03-22

**Authors:** Xavier C. E. Vrijdag, Hanna van Waart, Rebecca M. Pullon, Chris Sames, Simon J. Mitchell, Jamie W. Sleigh

**Affiliations:** 1 Department of Anaesthesiology, School of Medicine, University of Auckland, Private bag 92019, Auckland, 1142 New Zealand; 2 Department of Anaesthesia, Waikato Hospital, Hamilton, 3240 New Zealand; 3 Slark Hyperbaric Unit, Waitemata District Health Board, Auckland, 0610 New Zealand; 4 Department of Anaesthesia, Auckland City Hospital, Auckland, 1023 New Zealand

**Keywords:** Occupational health, Cognitive control, Consciousness

## Abstract

Divers commonly breathe air, containing nitrogen. Nitrogen under hyperbaric conditions is a narcotic gas. In dives beyond a notional threshold of 30 m depth (405 kPa) this can cause cognitive impairment, culminating in accidents due to poor decision making. Helium is known to have no narcotic effect. This study explored potential approaches to developing an electroencephalogram (EEG) functional connectivity metric to measure narcosis produced by nitrogen at hyperbaric pressures. Twelve human participants (five female) breathed air and heliox (in random order) at 284 and 608 kPa while recording 32-channel EEG and psychometric function. The degree of spatial functional connectivity, estimated using mutual information, was summarized with global efficiency. Air-breathing at 608 kPa (experienced as mild narcosis) caused a 35% increase in global efficiency compared to surface air-breathing (mean increase = 0.17, 95% CI [0.09–0.25], p = 0.001). Air-breathing at 284 kPa trended in a similar direction. Functional connectivity was modestly associated with psychometric impairment (mixed-effects model r^2^ = 0.60, receiver-operating-characteristic area, 0.67 [0.51–0.84], p = 0.02). Heliox breathing did not cause a significant change in functional connectivity. In conclusion, functional connectivity increased during hyperbaric air-breathing in a dose-dependent manner, but not while heliox-breathing. This suggests sensitivity to nitrogen narcosis specifically.

## Introduction

Divers breathe compressed gases at increased environmental pressures^1^. Most commonly, this is air, consisting of 21% oxygen and 79% nitrogen, sometimes substituted with varying fractions of oxygen or helium^2^. Each of these gases has physiological effects, dependent on the gas's partial pressure^3^.

An increased partial pressure of nitrogen is known to cause cognitive impairment proportional to the inspired pressure, also known as ‘nitrogen narcosis’. This effect is considered to be noticeable at and beyond 30 m of seawater (msw) (405 kPa) when breathing air^4^. Breathing air at 608 kPa is typically cited as causing sleepiness, euphoria, overconfidence, idea fixation and impaired reasoning, memory, calculus and judgement. However, these symptoms may be ameliorated by enhanced concentration in motivated subjects^5^. At this level, nitrogen narcosis is not directly hazardous to the diver, but may predispose to erroneous decisions that can lead to an incident or accident. To lower the gas mixture's narcotic potency for deeper diving, divers can partially or totally substitute nitrogen with helium, a non-narcotic but expensive gas^2^.

Quantification of the subtle effects of inert gas narcosis is challenging, particularly in real-time during diving. Psychometric testing of higher cognitive functions like memory, inhibition and learning^6^ show increased reaction time and errors with nitrogen narcosis^7^. However, psychometric tests can be confounded by learning effects, participant motivation or boredom^8^; and the tests interfere with diving activities, making them unsuitable for continuous monitoring.

Objective neurophysiological measurements could, in principle, be used to overcome these issues. For example, quantitative EEG (qEEG) analysis could monitor the diver continuously, without interfering with diving activities. EEG can be analyzed with a wide variety of methods, but none have yet been successfully applied to measuring nitrogen narcosis.

A common qEEG method is frequency analysis. Unfortunately, it does not provide the sensitivity needed to accurately measure narcosis at levels commonly seen in diving, as significant changes in frequency power are only found at far higher pressures^9–17^. Functional connectivity metrics are more sophisticated qEEG methods which have been shown to be sensitive to changes in consciousness^18–27^ caused either by disease (minimal conscious state or vegetative state) or anesthesia (propofol or sevoflurane). However, none of these metrics have been used to measure the relatively mild effects of gas narcosis, such as seen in diving.

Mutual information is a non-linear model-free measure^28,29^, that quantifies the overlap of information between signals, increasing sensitivity to capture neuronal processes, which are non-linear in nature^30^. The output is an enormous 2-dimensional functional connectivity matrix between all channels, which is hard to interpret. However, connectivity results can be summarized with graph theory methodologies, to quantify the changes in the connectivity of a network^31^. It has been suggested that a neuronal network needs an optimal balance between integration and segregation to function fully and share information effectively between brain regions^30^. Global efficiency is a related measure of node integration; highly efficient networks have all nodes connected, making information sharing easy. However, completely connected networks can become ‘saturated’, and have low complexity; making higher-order brain responses to internal and external environmental inputs impossible. Therefore a certain level of segregation is probably needed for optimal cognition^32^. Excessive integration and loss of segregation (i.e. increased network global efficiency) has been observed with hallucinogenic drugs.

The aim of this study was to develop a sensitive measurement tool for monitoring nitrogen narcosis in divers. It was hypothesized that—when compared to air-breathing at 101 kPa—breathing air at 608 kPa would cause cognitive impairment and increase mutual information amongst cortical regions, as summarized using global efficiency—similar to the effects of hallucinogens. On the other hand, it was expected that breathing an oxygen-helium (heliox) mixture at 608 kPa would cause neither cognitive impairment nor an increase in mutual information.

## Results

The twelve divers (5 female), aged between 24 and 55 years (mean 36 years ± 11), with an average body mass index of 26.7 kg/m^2^ (± 3.2), had made on average 921 dives [160–2000] over 15 years [3–28]. Seven divers had dived below 50 msw (608 kPa) using air. For brevity, only baseline (101 kPa) and 608 kPa comparison results are reported unless a significant effect was found, in which case the 284 kPa results were added to the analysis to ascertain if there was a dose–response pattern.

No adverse events occurred on any exposures. There was no evidence of CO_2_ retention in any subjects on the basis of end-tidal CO_2_ measurements made at 284 kPa during decompression.

### Psychometric test

**Figure 1:**
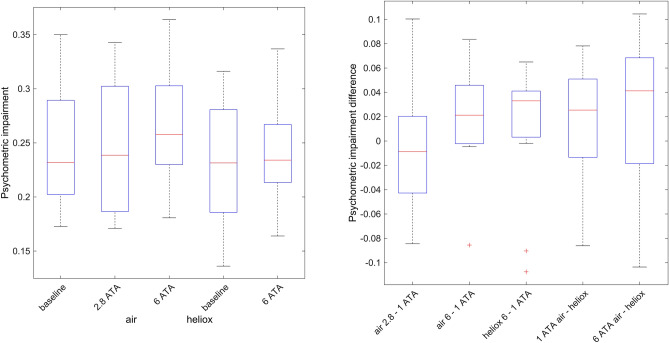
Box plot of the psychometric test results (reaction time and accuracy) combined metric (left) and the within-subject differences between exposures (right). The box plot shows the median (red line), interquartile range (blue box) and the whiskers showing the full range of the data. Outliers (red +) are values larger than 1.5 times the interquartile range.

The psychometric test was not very sensitive to impairment caused by the hyperbaric air exposures, showing only a small increase in impairment (mean difference of 0.018, 95% CI − 0.007 to 0.043, p = 0.14) at 608 kPa. As expected, there was no change while breathing heliox (Fig. 1).

### Task load and sleepiness questionnaires

The NASA task load index scales for *mental demand* and *physical demand* were significantly increased between baseline and 608 kPa during exposures to both air (mean difference in *mental demand* of 10.8 (95% CI 2.5 to 19.2) and in *physical demand* of 5.4 (95% CI 0.8 to 10.0), p = 0.02 and p = 0.02) and heliox (mean difference in *mental demand* of 8.8 (95% CI 1.7 to 15.8) and in *physical demand* of 12.1 (95% CI 3.9 to 20.3), p = 0.02 and p = 0.001).

The Karolinska sleepiness scale questionnaire results were significantly different between the baseline and 608 kPa heliox exposures (mean difference of 1.0 (95% CI 0.05 to 1.95), p = 0.04) and between the heliox and air exposures at 608 kPa (mean difference 1.3 (95% CI 0.1 to 2.5), p = 0.03).

### Frequency power analysis

On average, 2.5 of the 30 2-s EEG samples were removed due to artefacts, mainly muscle and eye movement artefacts. Compared to heliox, at 608 kPa air the non-parametric cluster-based permutation testing indicated a significant cluster of decreased power in the delta and theta frequency bands in the bilateral frontal, midline and occipital electrodes (Cohens’ d = 1.15, p = 0.002).

**Figure 2:**
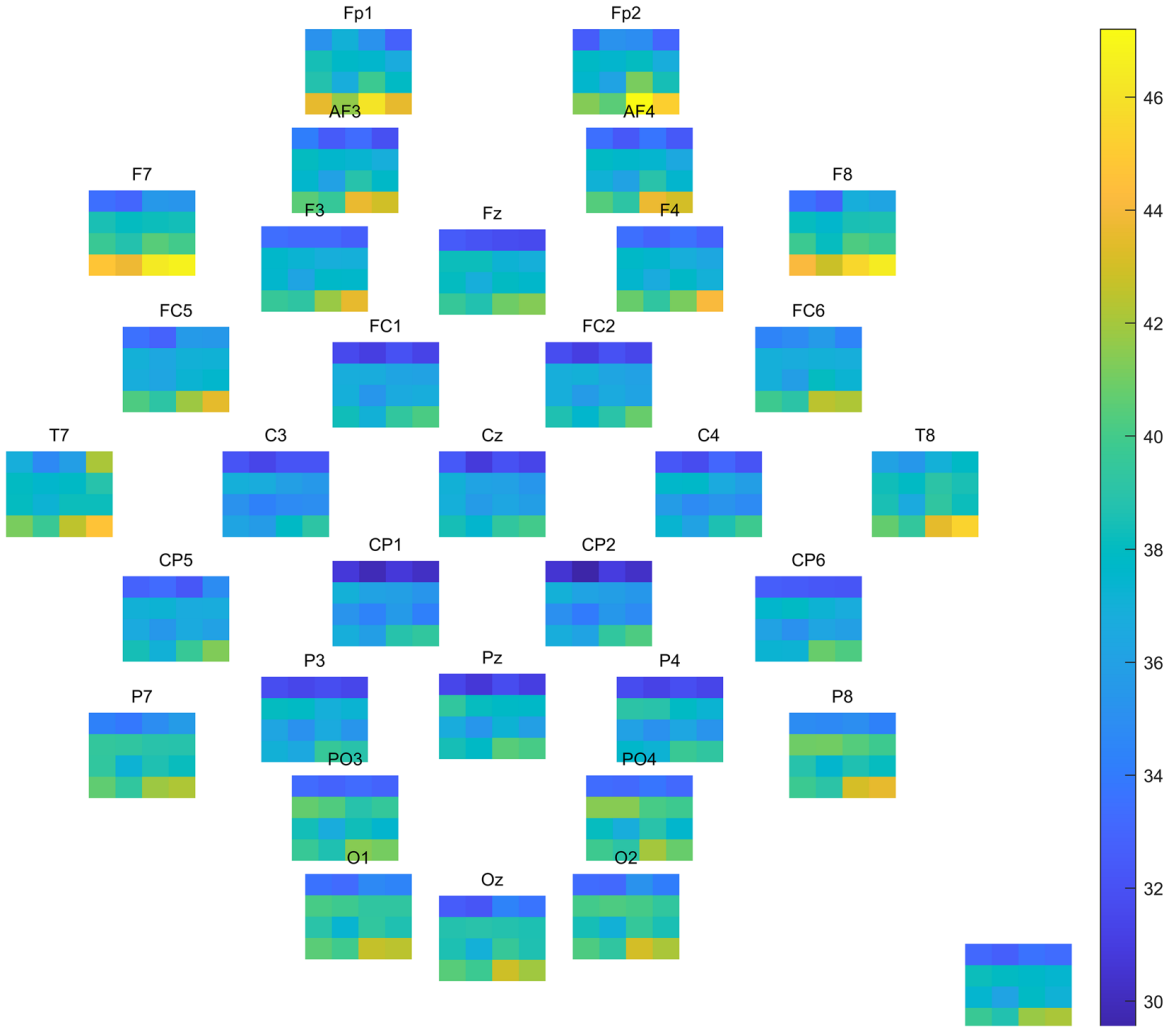
Average power in dB for each electrode (placed according to their head map location, title is the electrode name) and the mean of all electrodes (bottom right) for all participants. Each subfigure has the four exposures on the x-axis (air baseline, air 608 kPa, heliox baseline, heliox 608 kPa) and four frequency bands on the y-axis (bottom to top: delta (1–4 Hz), theta (4–8 Hz), alpha (8–14 Hz) and beta (14–30 Hz)).

When comparing baseline (101 kPa) and 608 kPa air exposures, there were two significantly different clusters based on the non-parametric cluster-based permutation testing, indicating a decrease in power in the delta and theta frequency bands, scattered over the electrodes (average Cohen’s d = 1.15, p < 0.001 and p = 0.049). Power in the alpha and beta band did not change. As expected, breathing heliox caused no change between baseline (101 kPa) and 608 kPa in any of the four frequency bands (Fig. 2).

### Functional connectivity

Compared to baseline (101 kPa), air breathing at 608 kPa caused significant *increases* in functional connectivity.

#### Pair-wise mutual information in the alpha frequency band

Air-breathing at 608 kPa significantly *increased* pair-wise channel mutual information compared to baseline in six clusters (p = 0.002–0.049, average Cohen’s d = 2.33, cluster-based permutation testing). These were mainly fronto-parietal oriented connections (Supplementary Figure S1). There was no significant difference in mutual information between the heliox exposures (101 vs 608 kPa).

**Figure 3:**
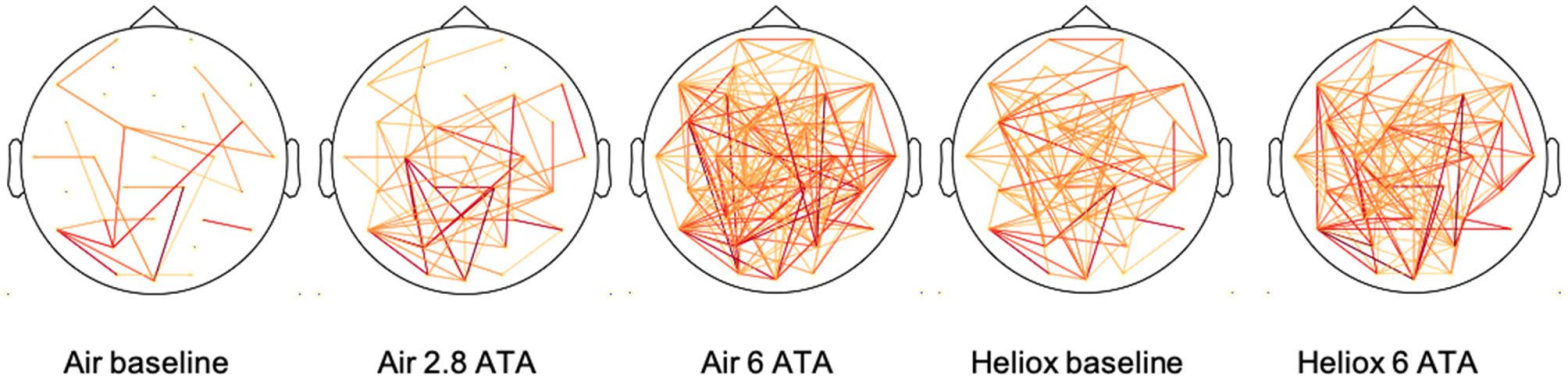
Head plots of binarized mutual information connections for each exposure. Yellow lines are common connections in more than three participants, while orange and red (> 9 participants) depict connections seen in most of the participants.

Similarly, the networks of binarized mutual information became more dense when breathing air at higher pressure (Fig. 3). At baseline (101 kPa) during air breathing, only four connections were highly common between participants (more than nine participants, seen in red). The number of highly common connections increased dramatically at higher pressure (to 22 at 284 kPa, and 40 at 608 kPa). Heliox breathing also started with four common connections at 101 kPa and increased much less to a mere twelve highly common connections at 608 kPa.

#### Network global efficiency

**Figure 4:**
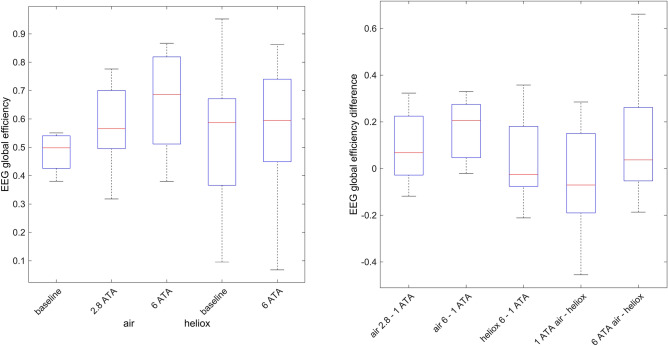
Box plot of the EEG mutual information based global efficiency metric (left) and the differences between exposures (right). The box plot shows the median (red line), interquartile range (blue box) and the whiskers showing the full range of the data.

Increased nitrogen partial pressure progressively increased global efficiency of the network. During air-breathing at 284 kPa and 608 kPa, the summary network statistic global efficiency showed a 19% and 35% increase compared to baseline (101 kPa). [Within-subject mean increase of 0.09 (95% CI − 0.00 to 0.18, p = 0.05) and 0.17 (95% CI 0.09 to 0.25, p = 0.001) respectively]. Between the baseline and 608 kPa heliox exposures, there was no significant difference. Comparing air and heliox at 608 kPa, there was a non-significant mean difference of 0.10 (95% CI − 0.06–0.25, p = 0.20) (Fig. 4).

#### Global efficiency based linear mixed-effects modelling

**Figure 5:**
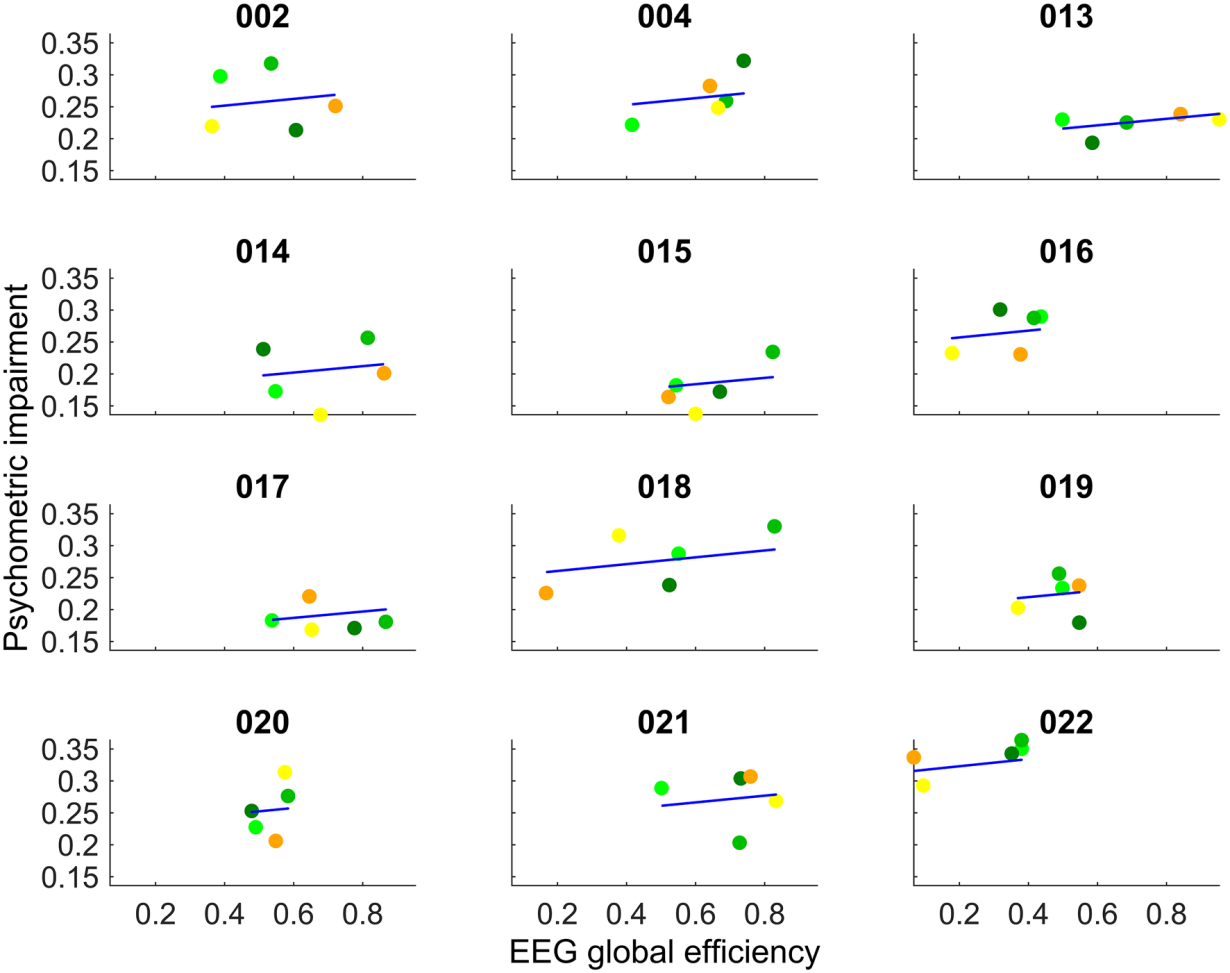
EEG mutual information based global efficiency metric versus psychometric impairment for each subject. Colored dots depict each exposure (light green = air baseline, middle green = 284 kPa air, dark green = 608 kPa air, yellow = heliox baseline and orange = 608 kPa heliox). Blue lines are the fitted model.

**Figure 6:**
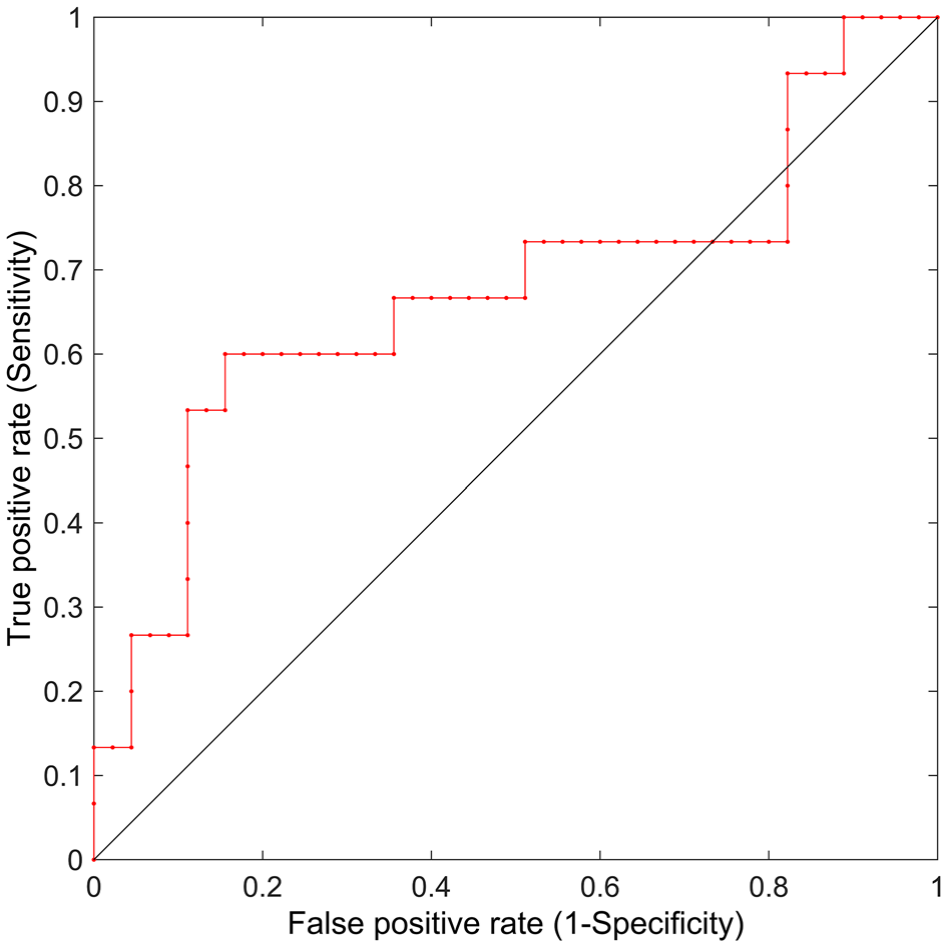
Receiver operating characteristic (ROC) graph comparing the sensitivity and specificity of the EEG mutual information based global efficiency metric versus the psychometric test results.

There was a positive slope in the linear mixed effect modelling between global efficiency and psychometric impairment for all participants, indicating that increased global efficiency was tracking psychometric deterioration for all participants (Fig. 5). The linear mixed effect model showed a significant, but modest fit (r^2^ = 0.60, AUROC = 0.67 (95% CI 0.51 to 0.84, p = 0.02)) (Fig. 6 and Supplementary figure S2).

## Discussion

This study explored potential approaches to developing a quantitative EEG metric to measure narcosis produced by nitrogen at hyperbaric pressures. The global efficiency metric based on mutual information was able to differentiate between surface air-breathing and air at 608 kPa; the results at the much lower pressure of 284 kPa were intermediate. This indicates a dose–response between functional connectivity (as measured with global efficiency) and nitrogen narcosis. No significant change in the metric was found during the hyperbaric heliox exposures, confirming that the effects of nitrogen exposure are induced by the breathing gas and not by environmental (pressure) changes per se. Only a moderate relation between the EEG metric and psychometric impairment was found with the linear mixed effect model. This can be attributed to the psychometric test's relative insensitivity to narcosis caused by hyperbaric air.

### Functional connectivity

In this study, breathing air at increased pressure caused an *increase* in mutual information in the alpha frequency band. The causes of this phenomenon are not well understood, but mathematical modelling of a thalamo-cortical network has indicated that during GABA-receptor inhibition, the network can oscillate in the alpha–beta frequency band^33^. This phenomenon has been described in animal studies with both cortical and thalamic electrodes during propofol anesthesia^34,35^. Clinically, propofol is known to induce synchronous alpha (and beta) oscillations during induction of anesthesia^36^, and several studies have confirmed this; both during slow propofol induction^37,38^, and also when using propofol to sedation levels only^27^. Hyperbaric nitrogen is known to potentiate signal transduction at the GABA-receptor as well^39,40^. Hence, it is plausible that the observed increase in functional connectivity as measured by mutual information can be explained by an increase of thalamo-cortical coupling and consequent cortical dysfunction.

Similarly, the integration of the network measured with global efficiency increases, indicating an increase in thalamic driving, which can occur during a decrease in cortico-cortical connectivity^27^. These cortical connections are needed for an integrated network where information can flow between brain regions to sustain higher-order cognitive functioning^41^. The increase in mutual information is predominantly concentrated in frontal hubs—connecting to a range of posterior nodes—which is known to support higher-order cognition^42,43^.

### Frequency power analysis

Hyperbaric air exposure caused a decrease in low-frequency band power and no change in the alpha band in this study. This contradicts previously described power and frequency changes^15–17^; however, those experiments were done at substantially higher exposure pressures (709 and 911 kPa).

### Subjective behavioral questionnaires

Participants found it more mentally and physically demanding to perform their tasks and felt sleepier at pressure compared to baseline. This result was the same for both gases, even though the participants were not blinded to the gas they were breathing. This suggests that the subjective grading of task performance is sensitive to depth per se, but insensitive to nitrogen narcosis. Because the questionnaires were not able to discriminate between heliox and air at 608 kPa, they can be considered to be unreliable for performance monitoring, consistent with prior reports that subjective reporting on narcosis is unreliable^44,45^.

### Strengths and limitations

In this study, the order of gases was randomized to account for a possible learning effect in the psychometric test. It was conducted inside a hyperbaric chamber, which strengthened the environmental control, making it very consistent between the experiments. It is unknown if the results can be translated to the underwater environment, which would require further investigation.

Only twelve participants were part of this study, but the changes in EEG global efficiency were consistent between all participants in this small group. The psychometric test's sensitivity was an issue, as only a slight cognitive deterioration was seen at 284 kPa and greater, and this was still not statistically significant while breathing air at 608 kPa. Previously, this same test has shown significant impairment at 405 kPa^46^. These inconsistent results show the problems faced in attempts to measure cognitive impairment. However, the EEG was capable of detecting a difference between the pressure exposures, potentially making it a more sensitive measure for narcosis than the psychometric test.

The linear mixed effect model showed a relation between the psychometric impairment and the EEG metric. Some participants (016 and 018 in Fig. 5), paradoxically exhibited greater impairment on the lower of the two nitrogen exposures. This may be explained by the varying focus, boredom and training effects on psychometric test performance between and within individuals. Furthermore, breathing air at 608 kPa is not a strongly debilitating exposure and we found, subjectively, that some participants were able to volitionally increase their focus on the task thereby suppressing the narcotic effects. Nevertheless, Fig. 5 suggests that the EEG metric positively correlates with psychometric performance which, at the inspired nitrogen pressures used here, may be inconsistently related to the nitrogen ‘dose’. We plan to address this in further work using a more sensitive psychometric test and a significantly greater nitrogen exposure (air breathing at 811 kPa), aiming to produce a degree of narcosis less amenable to modification by variable levels of concentration.

The current EEG pre-processing algorithm contains manual steps. In future developments of the algorithm an automated cleaning and selection algorithm will support the aim of a real-time monitoring algorithm.

EEG recordings suffer from volume conduction due to the tissue layers between the neuronal source and the skin electrodes^47^. This effect has been minimized by applying average referencing, and a Hjorth transformation was applied before the mutual information analysis.

Finally, we cannot definitively exclude CO_2_ retention at 608 kPa because of failure of our measurement device at this pressure. However, we saw no evidence of it at 284 kPa early in decompression from 608 kPa, and there are strong grounds for suggesting it was extremely unlikely. For example, in a study of resting divers at 679 kPa there was no evidence for CO_2_ retention, despite the additive risk of immersion, even when abnormally high breathing resistance was imposed^48^.

### Future directions

The global efficiency EEG metric based on mutual information has shown significant potential as an objective measure of nitrogen narcosis. Confirmation of the present findings, and improvement of the sensitivity and robustness of the metric could be achieved by recruiting larger numbers of subjects exposed to the same and increased pressures, to increase the narcotic effect. Replacing the psychometric test strategy with a more complex test is likely to increase the sensitivity for psychometric impairment. These steps will be implemented in ongoing studies as part of our research program (ANZCTR: U1111-1181-9722).

Ultimately, it is conceivable that a qEEG method could be used as a continuous real-time monitoring system that is wearable underwater in a diving helmet, creating a dry space. This system could warn the diver and supervisor of potential cognitive impairment, and as a result, reduce the chance of a serious diving accident.

## Conclusion

In conclusion, this exploratory study has shown that a network metric of functional connectivity distinguishes air-breathing at 608 kPa from a 101 kPa baseline while remaining unchanged when breathing non-narcotic helium. These promising results are the first step in quantifying the narcotic effect of hyperbaric nitrogen using an electrophysiological measurement. Further optimization and validation are needed for building a robust monitoring method for nitrogen narcosis.

## Materials and methods

### Experimental design and participants

This randomized cross-over trial took place at the Slark Hyperbaric Unit, Waitemata District Health Board, from February to May 2019. The study protocol was approved by the Health and Disability Ethics Committee, Auckland, New Zealand (reference 16/NTA/93) and was registered with the Australian New Zealand Clinical Trial Registry (ANZCTR: U1111-1181-9722). The research has been performed in accordance with the Declaration of Helsinki.

Participants were healthy divers (checked with the Recreational Scuba Council screening questionnaire for fitness), aged between 18 and 60 years, with normal visual acuity either corrected or uncorrected. They were certified technical divers trained to do decompression dives using oxygen as a decompression gas. Exclusion criteria were the current use of recreational drugs, tobacco, psychoactive medication, excessive alcohol (> 21 standard drinks per week), or over five daily caffeine-containing beverages. Participants had at least 6 h of sleep the night before, abstained from any caffeinated drink on the day and refrained from diving and alcohol 24 h prior to the commencement of each hyperbaric exposure. All participants provided written informed consent.

### Experimental procedures

All experiments took place in a cylindrical 5-person hyperbaric chamber (W.E. Smith Engineering PTY LTD, Australia). Participants completed two hyperbaric exposures at least 48 h apart; one breathing air and one breathing heliox (20.8% oxygen, balance helium) in random order, unblinded to the gas order. In the air sessions, participants underwent measurements at 284 kPa (equivalent to 18 msw depth) and 608 kPa (equivalent to 50 msw depth). In the heliox sessions, participants breathed heliox for measurements at surface pressure and at 608 kPa. The 284 kPa pressure was omitted during the heliox exposure to minimize decompression. Decompression was conducted according to the Defence and Civil Institute of Environmental Medicine (DCIEM) decompression tables, including 100% oxygen breathing from 193 kPa (equivalent to 9 msw depth) to surface pressure.

**Figure 7:**
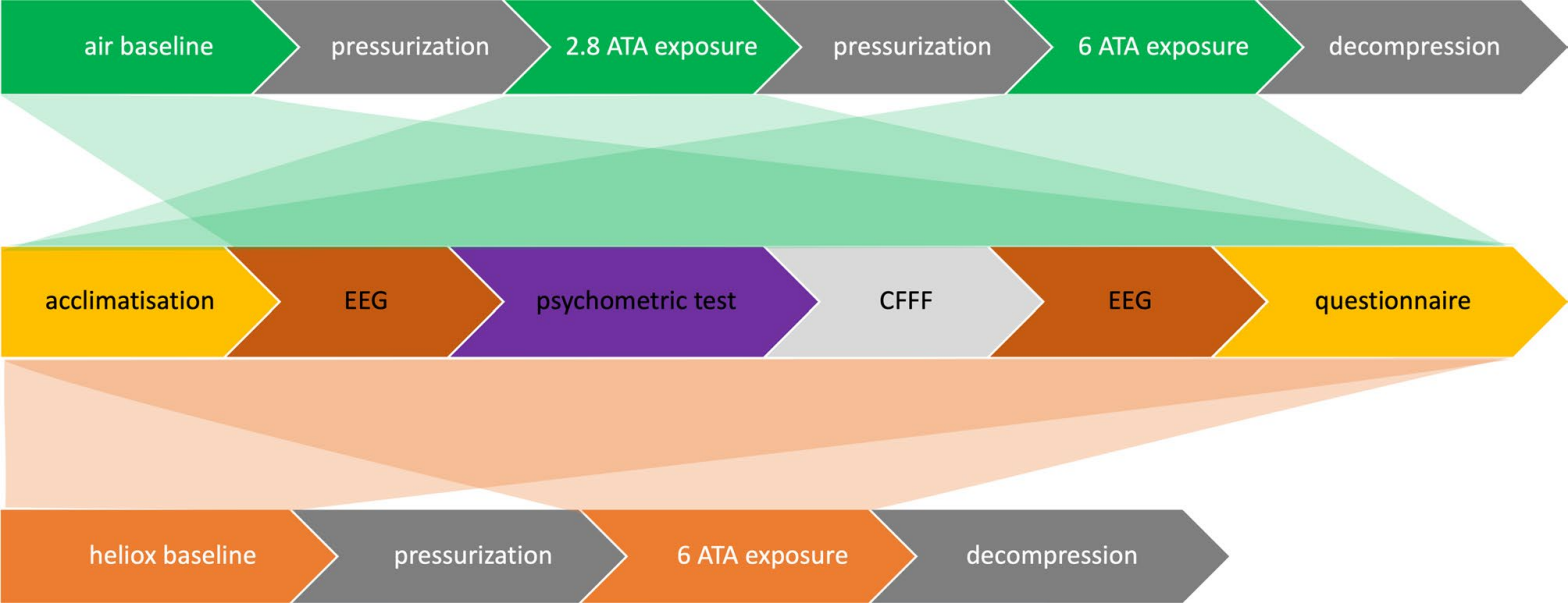
Air and heliox measurements. The top row describes the order of steps during the air measurement session. After preparation, a baseline measurement was conducted. Directly followed pressurization to 284 kPa, where the measurements were repeated. Further pressurization to 608 kPa was undertaken to repeat the measurements. The bottom row describes the heliox exposure, which involved measurements at the surface and 608 kPa. The middle row represents the recording order of the components in each measurement sequence after acclimatization. The air baseline measurement did not require an acclimatization step.

Every new gas/pressure exposure (see Fig. 7) started with a 5-min acclimatization period followed by a set of measurements consisting of: EEG recording over one-minute eyes open; one-minute eyes closed; psychometric tests and a critical flicker fusion frequency (CFFF) measurement^49^; repeat of the one-minute eyes-open and eyes-closed EEG recordings; and finally, the NASA task load index and Karolinska sleepiness scale questionnaires (Fig. 7). An attempt to measure end-tidal carbon dioxide (CO_2_) was made at 608 kPa using an in-line capnograph (EMMA, Masimo, Irvine CA, USA) but the device was unable to take readings beyond 557 kPa pressure. We therefore took readings to seek any evidence of CO_2_ retention on arrival at the first stop (284 kPa) during decompression.

### Outcomes

#### EEG recording

The EEG was recorded using a portable active electrode 32-channel system (ActiveTwo, BioSemi, Amsterdam, the Netherlands), in a sized cap based on the international 10–10 system^50^. Two additional electrodes were placed under the eyes to filter ocular artefacts. The offset (impedance equivalent for active systems) was checked for all electrodes, and electrode placement and gelling (SignaGel, Parker Laboratories, Fairfield, NJ, USA) were adjusted if the offset was above 25 µV. All signals were recorded at a sample rate of 1024 Hz using ActiView software (BioSemi) for offline analysis. For analysis, the resting-state eyes-open of the second EEG sample (after the psychometric tests, Fig. 7) was used, as a longer gas exposure preceded this measurement, and fewer artefacts were present. Moreover, the eyes-open state is a more natural state a diver would be in during their activities.

#### Psychometric test

The math processing test was selected from the psychology experimental building language (PEBL, RRID:SCR_014794) test battery^51^, which has shown sensitivity to subtle narcotic effects as it tests for higher-order cognitive functions like long-term and working memory, arithmetic operations and numeric comparisons (decision-making)^52^. The participant added and/or subtracted three one-digit numbers and had to decide if the result would be higher or lower than five within 5 s, repeated twenty times on a diving computer (Icon, Mares s.p.A., Italy) which stored reaction time and error rate. Participants attended a training session to mitigate a learning effect during the actual measurements by practicing until stable results were achieved.

#### NASA task load index and Karolinska sleepiness scale

The NASA task load index measured subjective experience of work^53^ on six scales; mental demand, physical demand, temporal demand, performance, effort, and frustration level. The Karolinska sleepiness scale questionnaire measured subjective wakefulness level on a scale from 1 (extremely alert) to 9 (very sleepy)^54^. The raw NASA task load index test, which omits the weighting questions^55^, and the Karolinska sleepiness scale were administered on paper.

### Analysis

The EEG data were pre-processed and then analyzed with frequency analysis and connectivity analysis algorithms.

#### EEG pre-processing

The EEG data were cleaned using the Fieldtrip toolbox (version c6d58e9 RRID:SCR_004849)^56^. The data for exposure were cut out of the continuous recording, re-referenced to the average, de-meaned, de-trended, and resampled to 256 Hz. Line (including higher harmonic) and low-frequency noise (< 1 Hz) were filtered out. Next, independent component analysis was used to filter noise components like eye blinks, high-frequency noise, non-physiological noise, and bad channels. An algorithm was used to advise on the manual selection of components. The data were cut in two-second epochs and manually inspected for remaining artefacts, with an algorithm indicating bad segments for remaining eye blinks (correlation with the EOG channels) and muscle artefacts (based on high-frequency content of 105 to 120 Hz, as this is mainly free of EEG signal and still contains EMG signal). Whole two-second epochs were discarded if they were marked as containing artefacts. The remaining epochs for that condition and exposure were stored for further analysis. See Vrijdag et al. (2021) for the script^57^.

#### Frequency power analysis

Frequency power was estimated using a multitaper Fourier transformation implemented in the Fieldtrip toolbox using a Hanning window from 1 to 100 Hz with 1 Hz increments. The average power per frequency band was calculated for: delta (1–4 Hz), theta (4–8 Hz), alpha (8–14 Hz) and beta (14–30 Hz) for each exposure.

#### Mutual information

**Figure 8:**
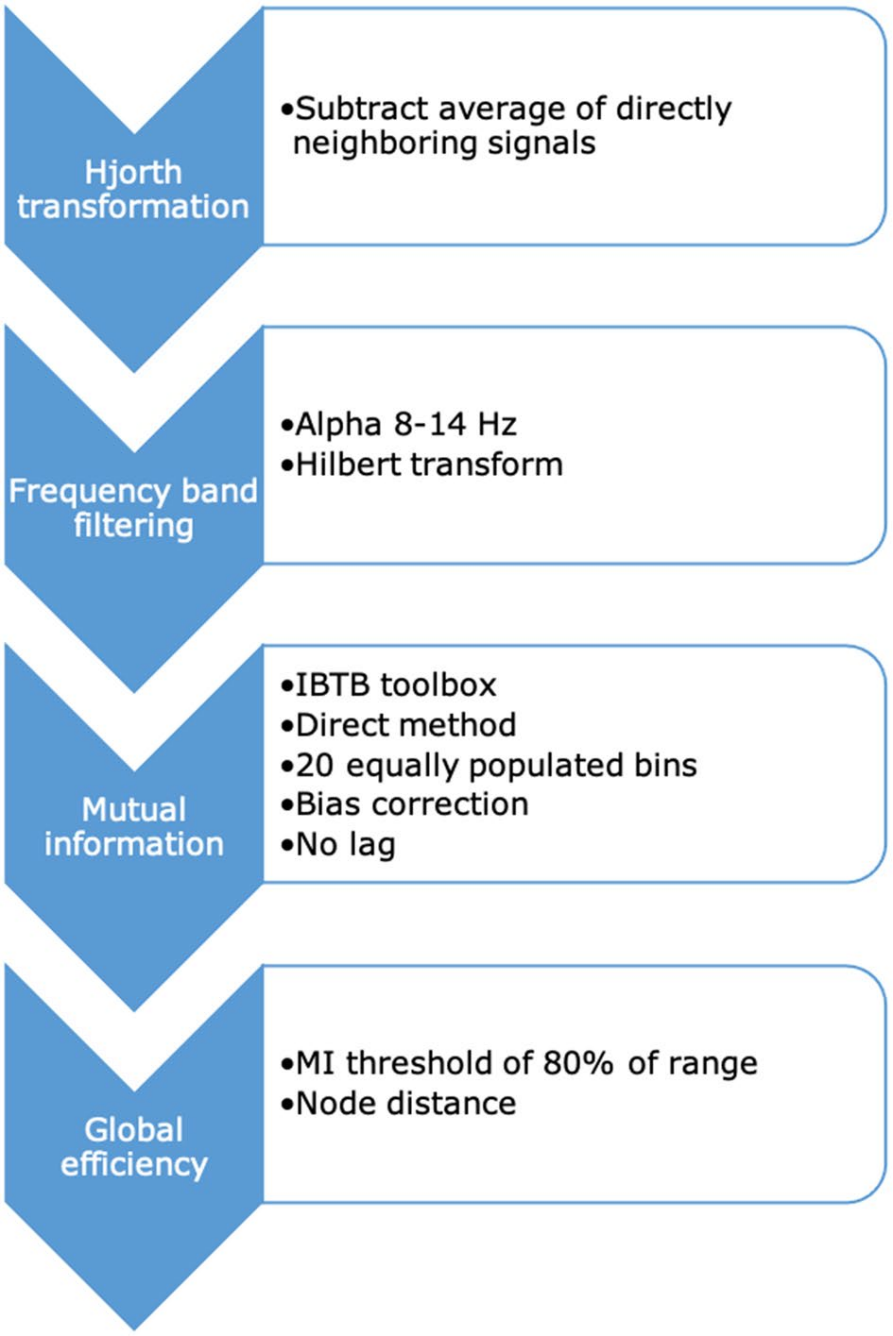
Flowchart of steps in the mutual information analysis.

After re-referencing using a Hjorth derivation, narrowband filtering (alpha band) and Hilbert transform, functional connectivity between the 32 channels was calculated using the mutual information direct method^58^ with 20 equally populated bins and bias correction^59^ as implemented in the information breakdown toolbox (IBTB) incorporated in Fieldtrip^58^. The connectivity matrix was binarized based on an individual threshold of 80% of the range of the 101 kPa air baseline matrix^60^. Global efficiency was calculated as summary network statistic, based on the node distance, also implemented in the fieldtrip toolbox^61^. See Fig. 8 and Supplementary material 3 for the script implemented using the Fieldtrip toolbox.

#### Psychometric test analysis

Data cleaning consisted of removing outlier test results where the reaction time was 0 ms. The two slowest test results were removed to counteract inattention^62^. Mean reaction time and error rate were calculated based on the results of the remaining tests. A combined 'psychometric-impairment' metric (scaled 0 to 1) was calculated using both the mean reaction time and error rate to counteract the speed-accuracy trade-off (Eq. 1)^63^.

1 $$\frac{\frac{mean\, reaction\, time}{\mathit{max}\,response\, time} + \frac{number\, of\, errors}{number\, of\, questions}}{2}$$

#### NASA task load index analysis

The ticks on each of the scales were digitized by rounding up to the nearest increment of five on a 0 to 100 scale^64^.

### Statistical analysis

All data were analyzed with Matlab version 2018a (Mathworks, Natick, MA, USA) except the psychometric test results, the NASA task load index and the Karolinska sleepiness scale, which were analyzed with SPSS version 27 (IBM, Armonk, NY, USA). All outcome measures were tested for normality with the Kolmogorov–Smirnov (K-S) test, and subsequently characterized by their mean and standard deviation (SD). Changes in functional connectivity were assessed at two levels: whole-scalp network summary statistics and pair-wise channel comparisons. Comparisons of global efficiency, psychometric test results, the NASA task load index and the Karolinska sleepiness scale between the exposures were analyzed with a two-tailed paired t-test and reported as mean difference and 95% confidence intervals (CI). Descriptive statistics were generated to characterize the study participants. p-values were regarded significant at p < 0.05, with Bonferroni correction for multiple comparisons.

#### Cluster-based permutation testing

To evaluate the topographic patterns of significant differences between exposures in average frequency band power, and pair-wise channel mutual information functional connectivity between all 32-by-32 channels, we used cluster-based permutation testing, as implemented in the Fieldtrip toolbox^65^. This procedure has the advantage of controlling for multiple comparisons in two steps; first using clustering methods, and second by creating a surrogate null distribution which is used to compare the obtained cluster data to determine p-values^66^.

In the first step, adjacent spatiospectral points were clustered according to which dependent t-values exceeded the cluster threshold p-value of 0.05 (two-tailed). The sum of the t-values for each cluster was calculated for the cluster-level statistic. The cluster-level statistics were evaluated under a randomized null distribution of the maximum cluster-level statistics, to control for the type I error rate.

For the second inference step, the randomized null distribution of maximal cluster-level statistics for chance was obtained by randomly shuffling the condition labels 1000 times. At each of these randomizations, the cluster-level statistic was computed, and the largest statistic was entered into the null distribution. Finally, the observed cluster-level statistic was compared against the null distribution, and clusters with a p-value below 0.05 (two-tailed) were considered significant. Effect size (Cohen’s d) was calculated as an average over all significant cluster nodes.

#### Linear mixed-effects modelling

A linear mixed-effects model was used for all exposures and individuals to calculate the relationship between the EEG metric (*EEG*) and the psychometric test metric (*PM*) using Eq. (2).

2 $$PM \sim 1 + EEG + (1 + EEG | participant)$$

The between-participant variation was included as a random effect, with a random intercept and slope. This design can capture variation in baseline global efficiency and dose–response for each subject.

A receiver-operating-characteristic analysis was used to compare the EEG metric against the psychometric test results. The psychometric test data were dichotomized to 'impaired' and 'not-impaired', based on the upper value of the interquartile range of the baseline exposure.

## Supplementary Information

Supplementary Information 1.

Supplementary Information 2.

Supplementary Information 3.

## Data Availability

The EEG data that support the findings of this study are available from the corresponding author upon reasonable request with a data-sharing agreement. The software code has been shared with this manuscript.
