# Supplementary material for: EEG functional connectivity is sensitive for nitrogen narcosis at 608 kPa
Source: Sci Rep. 2022 Mar 22;12:4880. doi: 10.1038/s41598-022-08869-8 (PMC8940999; doi:10.1038/s41598-022-08869-8)
Supplement: Supplementary file 1 — Supplementary Information 1. [file 41598_2022_8869_MOESM1_ESM.pdf]

Supplementary Figure S1

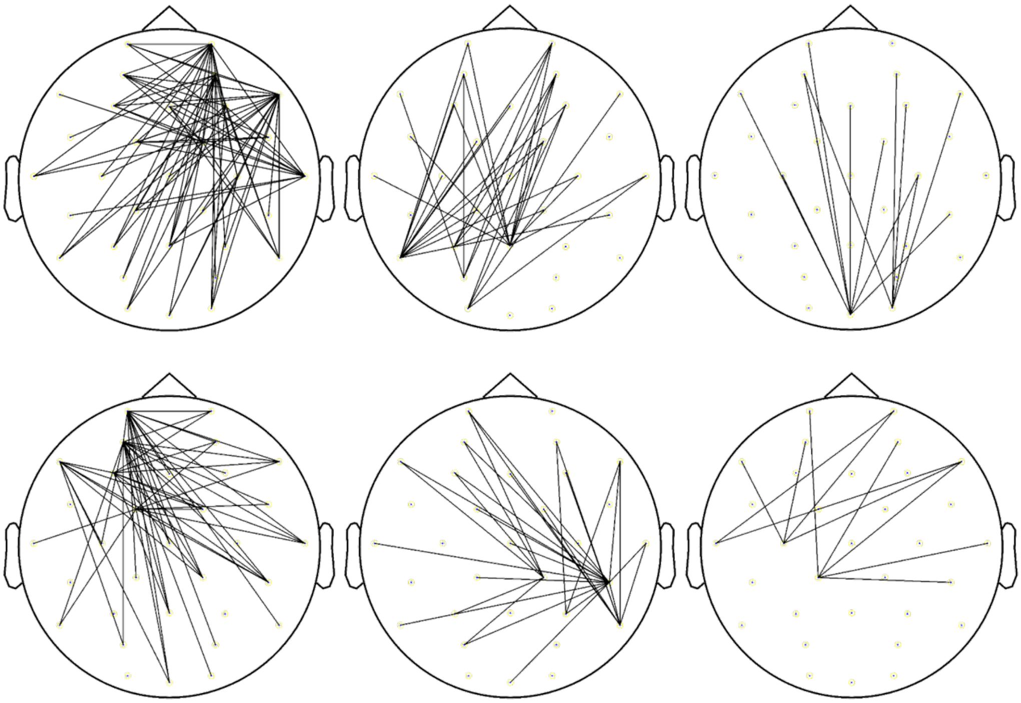

Six significant clusters of connections between EEG channels. Data (filtered between 8-14 Hz) is from the permutation testing, comparing 608 kPa of air-breathing and its baseline (101 kPa) measurement.
