# Supplementary material for: EEG functional connectivity is sensitive for nitrogen narcosis at 608 kPa
Source: Sci Rep. 2022 Mar 22;12:4880. doi: 10.1038/s41598-022-08869-8 (PMC8940999; doi:10.1038/s41598-022-08869-8)
Supplement: Supplementary file 2 — Supplementary Information 2. [file 41598_2022_8869_MOESM2_ESM.pdf]

Supplementary Figure S2

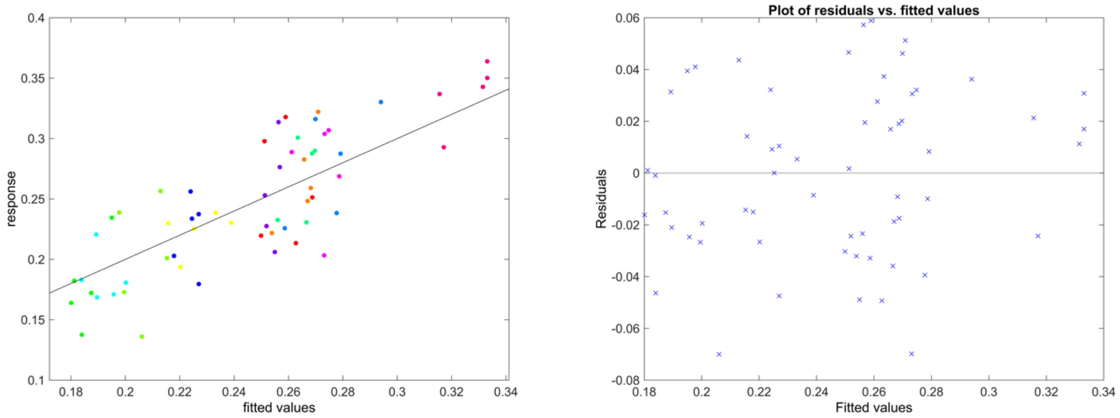

Graphs showing the results of the linear mixed effect model based on the EEG mutual information-based global efficiency metric versus the psychometric test results (response). Left panel shows the estimated psychometric test values based on the EEG metric (from the model) versus the actual psychometric test results. Each color represents the data of a single participant. Right panel shows the fitted results versus the residuals.
