# Supplementary material for: EEG functional connectivity is sensitive for nitrogen narcosis at 608 kPa
Source: Sci Rep. 2022 Mar 22;12:4880. doi: 10.1038/s41598-022-08869-8 (PMC8940999; doi:10.1038/s41598-022-08869-8)
Supplement: Supplementary file 3 — Supplementary Information 3. [file 41598_2022_8869_MOESM3_ESM.pdf]

Supplementary material 3: Connectivity analysis algorithm

```
close all
clear
clc

addpath(genpath('fieldtrip-fieldtrip-c6d58e9/external/ibtb')) % all
subdirectories of toolbox are needed)
load ('biosemi32elec_aligned.mat')
load('elecgroup');

[datasetselection,datapath] = data_selector;
epochtype= 'open end';
%exposures={'baseline' '20%' '30%' '40%' 'final'};
exposuresN2={'baseline' '2.8 ATA' '6 ATA'};
exposuresHe={'baseline' '6 ATA'};
exposuresO2={'baseline' '1 ATA' '1.4 ATA' '2.8 ATA'};

freqs= [8 14];%
binwidth=0.02;%Binwidth for MI calcs

cfg                 = [];
cfg.method          = 'template';
cfg.template        = 'biosemi32_neighb.mat';
cfg.layout          = 'biosemi32.lay';
neighbours = ft_prepare_neighbours(cfg);

%% combining multiple measurements per participant
for i=1:length(datasetselection)
    dataset_toks(i,:)=tokenize(datasetselection{i});
end
participants=unique(dataset_toks(:,2));
gases=flip(unique(dataset_toks(:,3)));

for i=1:length(participants)
    idx=find(strcmp(participants(i),dataset_toks(:,2)));
    if strcmp(dataset_toks(idx(1),3),'N2')
        datasetsorted(i,:)=datasetselection(idx);
    else
        datasetsorted(i,:)=datasetselection(flip(idx));
    end
end

%%
MI_ibtb=[];
for k=1:size(datasetsorted,1) %all selected participants
    expoI=1;
    for l=1:size(datasetsorted,2) %all gas condidions
        filepath=[datapath datasetsorted{k,l} filesep 'cleaned' filesep];

        participantnr=datasetsorted{k,l}(8:10);
        gas=datasetsorted{k,l}(12:13);

        switch gas
            case 'N2'
                exposures=exposuresN2;
            case 'He'
                exposures=exposuresHe;
            case 'O2'
```

```
                exposures=exposuresO2;
        end



        for m=1:length(exposures)
            filename=['CLEAN' datasetsorted{k,1} ' ' exposures{m} ' '
epochtype '.mat'];
            load([filepath filename])

            data=cleandata;

            %concatenate the trials into 1 continuous dataset
            data.trial={cell2mat(data.trial)};
            data.time={(0:(length(data.trial{1,1})-1))/data.fsample};

            % select EEG channels
            cfg            = [];
            cfg.channel    = 'EEG';
            data = ft_selectdata(cfg, data);

            %Hjorth transformation
            cfg            = [];
            cfg.method     = 'hjorth';
            cfg.elec       = elec_aligned; %from file
            cfg.neighbours= neighbours;
            datahj = ft_scalpcurrentdensity(cfg, data);

            %freqband + Hilbert analysis
            cfg            = [];
            cfg.bpfilter   = 'yes';
            cfg.bpfreq     = freqs;
            cfg.bpfiltord = 3;
            cfg.bpfilttype= 'but';
            cfg.bpfiltdir = 'twopass';
            cfg.hilbert    = 'abs';
            datafreq= ft_preprocessing(cfg,datahj);

            %mutual information
            cfg            = [];
            cfg.method     = 'mi';
            cfg.mi.numbin = 20;
            cfg.mi.lags    = 0;
            MI_ibtb{k,expoI}= ft_connectivityanalysis(cfg, datafreq);

            expoI=expoI+1;
        end
    end
end

%% network analysis
cfg2           = [];
cfg2.parameter = cfg1.parameter;
cfg2.method    = 'distance';

cfg3           = [];
cfg3.parameter = 'distance';
cfg3.method    = 'global_efficiency';
```

```
for i=1:size(MI_ibtb,1) % participants
    MIna=MI_ibtb{i,1};
    cfg2.threshold =  quantile(MIna.mi(~isnan(MIna.mi)),0.80);
    for j=1:size(MI_ibtb,2) %exposures
        MIna=MI_ibtb{i,j};
        MIna.mi(isnan(MIna.mi))=0;
        statDis = ft_networkanalysis(cfg2, MIna);
        statGE = ft_networkanalysis(cfg3, statDis);
        global_efficiency(i,j)=statGE.global_efficiency;
        %DIMORD participant, exposure
    end
end
```
